# Editorial Correspondence

**Published:** 1864-03

**Authors:** 


### EDITORIAL CORRESPONDENCE.

WASHINGTON, Feb. 16, 1864.

Notwithstanding this city is so well known, some things relative to it may not be devoid of interest to the readers of the JOURNAL. In general terms, I must say, I am much and unpleasantly disappointed in it. Its site has nothing to recommend it. It stands in a basin, surrounded by hills of slight elevation. It is reached only at great inconvenience, and has no business or commercial importance. It can be defended from a foreign or domestic foe only at great expense and effort, and it seems to me that when the war is over, and we are a united and peaceful people once more, the question will force itself on the public mind—where is the proper place for the capital of the nation? The public buildings—erected at great cost—are scattered over the city in such a way as to detract much from their influence on the mind, and the private edifices are very poor, and form with these, an unpleasant contrast.

There are some objects of interest here, among which is the Smithsonion Institute, founded by a munificent bequest made to our government by an Englishman,—James Smithson,—his avowed object being the dissemination of knowledge among men. In making this disposition of his wealth, he exhibited much of true wisdom, and placed his name where it will become more and more conspicuous, as those of the great body of his cotemporaries that now surround him shall one after another fall into oblivion. It is especially designed to give opportunities for scientific investigation, and what it has accomplished in this way under the supervision of Prof. Henry is well known to the public. It has an extensive library—its museum contains an interesting collection of prepared birds and animals—of shells and geological specimens—and also curiosities of different savage tribes, that illustrate strikingly the stages of their progress in civilization. It has, too, some works of art—a collection of Indian portraits, and 'tis to be hoped this may soon be the only reminder we have left of this worthless race. There are some heads in plaster—Cuvier's the only noticeable one—it is grand—the finest configuration I ever looked upon. There are a number, not yet dead, who will only live in clay, who have taken the precaution to place their names on the pedestals, that there may be no mistake. There is a pedestal for Pierce, but the bust is gone—translated, no doubt. I have long thought he stood in danger of this accident. The Washington monument is a sad affair. At a distance it reminds me of a kiln for the manufacture of pottery. It is unfinished, and is likely to remain so—for though contributions continue to be taken for it, it is not supposed they much more than cover incidental expenses. I should not think the view from it would ever be pleasant, and with its present surroundings, it is dismal enough. It is now in the midst of a great cattle yard, and around its base I saw one or two hundred government cattle—ordained victims for the shambles, and near it are racks for feeding, sheds for sheltering the hay, a long low building filled with quarters of beef, and standing around it army wagons to convey it as needed to the camps. In a building resembling this, I found the stones sent by different nations, States, and associations, to be inserted in the monument. The inscriptions on some interested me. For example: "The State of Louisiana, ever faithful to the Constitution and Union," Let us hope this may soon not be inappropriate. Another seceding State has adopted the language of Jackson: "The Federal Union, it must be preserved." The American Medical Association has a contribution, with a design suggested by Davids' painting representing Hippocrates spurning the gold offered by Xerxes, if he would desert his own people and engage in the service of the king. The design is rendered peculiarly happy from the contempt in which the profession has always been known to hold money. In the figure, the hand with which he was motioning them back has been broken off—doubtless by some faithless wretch, who feared that professional virtue might not be always proof against the temptation, and that the treasure might be grasped.

There is nothing in Washington so interesting to the physician as the collection instituted by the Surgeon General—Dr. Hammond—designed to illustrate the medical and surgical history of the rebellion. At the last session, Congress appropriated $5,000 in aid of the undertaking, and double this sum is asked of the present, and I doubt not there is enough of enlightened wisdom in this body to secure the money. So far as I know, it is the first instance in which the general government has done anything to aid the profession, to which, especially in the present war, it is so much indebted. The museum contains about 5,000 preparations, both wet and dry. The osteological specimens show every conceivable injury liable to occur from the accidents of war, and passing through all the stages from recent injury to long protracted efforts at repair. They are beautifully mounted, and the collection is said, by those of extensive foreign travel, to be, in regard to recent injuries, superior to any in the world. The proportion of specimens where union has taken place is small, for such patients have recovered without amputation, and cannot yet spare their limbs for exhibition. The wet preparations include many beautiful specimens of intestinal disease, so common in the army—showing every stage and grade of ulceration, perforation, enlarged follicles, &c.,—also, scorbutic ulcers of the mouth and throat, extensive diphtheritic membranes, hospital gangrene, and other objects of interest that I cannot enumerate. Excellent drawings of pathological conditions are being executed in water colors, designed to furnish the plates for the medical and surgical history of the war, that the Surgeon General intends to publish. A large number of microscopic preparations, both pathological and physiological, are already made, and are every day being increased.

Previous to this time, we have always looked abroad for exhibitions of the highest perfection in this art—but already, the skill which is the fruit of constant practice has enabled us to equal the best efforts of the old world. There is also a laboratory in which the purity of samples of medicines furnished to the government are tested, and in the medical department we have to give thanks that the days of "shoddy" are past.

A series of observations are now being instituted, with a view of finding out, if possible, the occult causes, atmospheric or otherwise, which tend to the propagation of diseases most common in hospitals. This work is being carried on under the supervision of the Surgeon General, by Drs. Brinton and Woodward, and it could hardly be placed in better hands. They possess the requisite attainments, and pursue their labors with the ardor inspired by the love of scientific investigation. It is to be hoped nothing may interrupt their work, and that from their labors we may preserve something to medicine and surgery of the rich experience of the war.

I think the profession, and the army are under obligation to Dr. Hammond for what he has done as Surgeon General. His position has been one of exceeding difficulty. No man in this place could avoid giving offence to many—and especially would this be true, if he performed the duties of his station well. All who find themselves thwarted in their designs on the public, will curse the obstacle that stands in their way, and he who disturbs traditionary usages, however unreasonable, will surely be anathematized, and I suspect that when Gabriel summons the elect to the fruition promised as the reward of their virtues, the half of them will lose their temper at his disturbance of their slumbers.

Our peace establishment for the medical department hardly furnished a nucleus for what is demanded by this gigantic war. The Surgeon-General had everything to organize and supply, and the sanitary condition of the army attests how well he has performed this task. I think him particularly well fitted for his place at the head of the Medical Bureau. His scientific acquirements and practical professional ability are well known,but he superadds to these executive capacity, pride in the profession, a jealous watchfulness over its honor and interests, and an enterprise and activity in carrying out his designs, quite shocking to dull and slow routinists. His lack of subserviency to non-professional men, who so often think they know better than the members of the profession about medical matters, has given offence to some. The attacks on him have been pressed with vigor, and long continued, and his position has been made as unpleasant as possible, perhaps with the hope that he might be thus made to resign. I have long thought there was a systematic attempt being made to prejudice him in the public mind, by the use of the telegraph, and in this way during the last four months, we have had him, over and over again, court-martialed, suspended from duty, or dismissed from the service. For some cause he finds in the Secretary of War a very bitter enemy, who treated him in such a manner as to compel Dr. Hammond to ask a court-martial to inquire into his official conduct, which favor was obtained only after more than one request, and not until the appeal had been made to the well-known sentiments of justice of the President himself. No efforts will be spared by Mr. Stanton to be rid of Dr. Hammond. The court is now in session in this city, is composed of nine members, and is presided over by Maj. Gen. Oglesby of Ill. He is the only one of whom I have much knowledge, but the people of Illinois know that he is beyond the reach of political or personal influences, and would do intentional injustice to no man.

I designed my letter to be brief, but fear it has grown to an unpardonable length. E. I——.

---

☞ We owe an apology to many friends for not returning receipts promptly for money received on subscriptions. The delay has been unavoidable, but we hope to correct it with the April issue.

